# Diagnostic Accuracy of Urine-Based HPV Testing Compared With Clinician-Collected Cervical Samples for Cervical Intraepithelial Neoplasia Grade 2 or Worse (CIN2+) Detection: A Systematic Review and Meta-Analysis

**DOI:** 10.7759/cureus.95518

**Published:** 2025-10-27

**Authors:** Jeevika G, Meshael Shaikh, Suraj Palavilayil, Gabriela Fernandes

**Affiliations:** 1 Gynaecology, Saveetha Medical College, Thandalam, IND; 2 Rheumatology, Manipal Hospitals Bangalore, Bengaluru, IND; 3 Cardiology, Manipal Hospitals Bangalore, Bengaluru, IND; 4 Hospital Medicine, M S Ramaiah Medical College and Teaching Hospital, Bengaluru, IND; 5 Medical Education, Blackpool Teaching Hospitals NHS Foundation Trust, Blackpool, GBR; 6 Periodontology and Oral Biology, SUNY BUFFALO, School of Dental Medicine, Buffalo, USA

**Keywords:** human papilloma virus, meta-analysis, pap smear test, systematic review, urine hpv analysis

## Abstract

Cervical cancer remains a major global health challenge, and human papillomavirus (HPV) testing has become central to screening and prevention programs. Conventional clinician-collected cervical samples, while effective, face barriers of invasiveness, infrastructure requirements, and limited acceptability in certain populations. Urine-based HPV testing has emerged as a potential alternative, offering advantages in convenience and patient preference, but concerns regarding diagnostic accuracy remain. This systematic review and meta-analysis, conducted in accordance with Preferred Reporting Items for Systematic Reviews and Meta-Analyses (PRISMA)-diagnostic test accuracy (DTA) guidelines, evaluated the diagnostic accuracy of urine-based HPV testing for the detection of cervical intraepithelial neoplasia grade 2 or worse (CIN2+) compared with clinician-collected cervical samples. A comprehensive search of PubMed, Embase, Scopus, and the Cochrane Library from January 2010 to August 2025 identified five eligible studies comprising 1,031 participants. Data were pooled using a bivariate random-effects model. Urine HPV testing demonstrated a pooled sensitivity of 74% (95% confidence intervals (CI), 67-80%) and a specificity of 52% (95% CI, 43-61%). Performance improved significantly when first-void urine collection and PCR-based assays were employed, with sensitivity approaching that of self-collected vaginal samples. Cervical samples consistently outperformed urine samples in sensitivity, though patient acceptability was higher for urine testing, with over 90% of women preferring it. The included studies had generally low risk of bias, and the heterogeneity was primarily related to assay type and urine collection methods. These findings suggest that while urine HPV testing cannot yet replace cervical sampling in primary screening, it holds strong potential as a complementary strategy, not replacement, to improve screening uptake.

## Introduction and background

Cervical cancer is the fourth most common cancer in women globally, with around 660,000 new cases and around 350,000 deaths in 2022, according to the WHO [1]. Persistent infection with high-risk human papillomavirus (hrHPV) is the primary etiological factor for cervical carcinogenesis, and hrHPV testing has become the cornerstone of screening and prevention programs. Current screening strategies rely predominantly on clinician-collected cervical samples obtained during pelvic examination, which are subsequently tested for hrHPV DNA or cytology. While effective, this approach requires specialized clinical infrastructure, trained personnel, and patient compliance with invasive sampling procedures. These barriers contribute to under-screening, particularly in low-resource settings and among populations with cultural, social, or logistical challenges that limit clinic attendance.

To overcome these obstacles, alternative sampling strategies such as self-collected vaginal swabs and urine-based hrHPV testing have been investigated. First-void urine (FVU) samples, in particular, contain exfoliated cells and free HPV DNA originating from the genital tract, and technological advances in PCR-based assays have facilitated their reliable analysis [2]. Urine testing offers important advantages in terms of convenience, privacy, and patient acceptability, potentially increasing screening uptake. However, concerns remain regarding diagnostic accuracy compared with clinician-collected samples, especially for the detection of high-grade cervical intraepithelial neoplasia (CIN2+), the clinically relevant threshold for management.

Although multiple studies have evaluated urine hrHPV testing in women undergoing colposcopy or with abnormal cytology, findings have been inconsistent. Variability in urine collection methods (random urine vs. first-void), devices, and assay platforms has contributed to heterogeneous sensitivity and specificity estimates. While optimized first-void devices and PCR-based assays appear to improve diagnostic accuracy, no consensus has been reached regarding the clinical utility of urine-based testing in routine screening.

The objective of this systematic review and diagnostic test accuracy (DTA) meta-analysis was to synthesize available evidence on the performance of urine-based hrHPV testing compared with clinician-collected cervical samples for the detection of CIN2+. Specifically, the review aimed to quantify pooled sensitivity and specificity, evaluate heterogeneity by collection method and assay platform, and assess the potential role of urine hrHPV testing as an alternative or adjunct to conventional cervical screening.

## Review

Materials and methods

This systematic review and DTA meta-analysis were conducted in accordance with the Preferred Reporting Items for Systematic Reviews and Meta-Analyses of Diagnostic Test Accuracy Studies (PRISMA-DTA) guidelines. In addition, the observational studies included in this review were appraised according to the Strengthening the Reporting of Observational Studies in Epidemiology (STROBE) checklist. The protocol for this review was developed in advance and has been registered in the International Prospective Register of Systematic Reviews (PROSPERO) to ensure methodological transparency (CRD420251143872).

The study was structured according to the PICO framework to ensure clarity and reproducibility of the research question. The Population (P) included women undergoing cervical cancer screening; the Index test (I) was urine-based HPV testing; the Comparator (C) was clinician-collected cervical samples; and the Outcome (O) was the detection of CIN2+ or worse. Studies were considered eligible if they included women aged 18 years or older who were undergoing cervical screening or referred for colposcopy following abnormal cytology or hrHPV positivity. The index test of interest was urine-based hrHPV testing performed using any assay platform, including Hybrid Capture II, Cervista, Anyplex II, Roche Cobas 8800, or other PCR-based methods. Both FVU and random urine samples were eligible for inclusion. The comparator was clinician-collected cervical samples tested for hrHPV or histologically confirmed CIN2+, which served as the reference standard. The primary outcomes of interest were sensitivity and specificity of urine hrHPV testing for CIN2+ detection, with sufficient information required to construct 2×2 contingency tables of true positives, false negatives, false positives, and true negatives. Prospective, cross-sectional, and case-control diagnostic accuracy studies were eligible. Studies were excluded if they were reviews, editorials, case reports, or methodological-only studies, if they reported only the prevalence of urine hrHPV without CIN2+ verification, or if they did not provide sufficient diagnostic accuracy data to calculate sensitivity and specificity.

A comprehensive electronic search was performed in PubMed, Embase, Scopus, and the Cochrane Library for studies published between January 2010 and August 2025. The search strategy combined MeSH terms and free-text keywords, including “human papillomavirus,” “HPV,” “urine,” “urinary samples,” “self-collection,” “cervical intraepithelial neoplasia,” “CIN2+,” “diagnostic accuracy,” and “cervical cancer screening.” Boolean operators “AND” and “OR” were applied to refine results. The search was supplemented by screening reference lists of eligible articles and relevant reviews, and abstracts from major gynecology and oncology conferences were assessed to capture unpublished or ongoing studies. No language restrictions were applied. Screening software (Rayyan) was used, and the inter-rater agreement rate was(Cohen’s kappa = 0.82).

Study selection was performed in two stages. First, titles and abstracts were independently screened by two reviewers to identify potentially relevant articles. Full texts of eligible studies were then retrieved and assessed in detail against the inclusion and exclusion criteria. Disagreements at either stage were resolved by consensus or consultation with a third reviewer. The selection process was documented in a PRISMA flow diagram.

Data extraction was carried out independently and in duplicate by two reviewers using a piloted, standardized template. Information extracted included study characteristics such as author, year, country, design, population, sample size, and setting; details of the index test, including urine collection method, device used, assay platform, and positivity thresholds; the reference standard employed; and diagnostic outcomes, including sensitivity, specificity, and the 2×2 contingency data where available. Additional outcomes, such as patient acceptability, concordance (kappa values), and relative diagnostic performance between urine and cervical samples, were also recorded when reported. Discrepancies were resolved through discussion. In instances where diagnostic outcomes were incompletely reported, contingency data were reconstructed from reported sensitivity, specificity, and sample sizes using standard formulas.

The methodological quality of included studies was assessed using the QUADAS-2 tool (Robvis), which evaluates four domains: patient selection, index test, reference standard, and flow/timing. Each domain was classified as having low, high, or unclear risk of bias. Results of this appraisal were presented graphically in both a traffic-light style plot and a bar-graph summary of proportions. Given the small number of included studies (n=5), formal testing for publication bias (e.g., Deeks’ funnel plot) was not feasible.

Diagnostic accuracy measures from each study were extracted as true positive, false negative, false positive, and true negative values. Pooled sensitivity and specificity were calculated using a random-effects univariate meta-analysis model to account for between-study variability. Separate meta-analyses were performed for sensitivity and specificity, and results were presented as pooled point estimates with corresponding 95% confidence intervals (CIs). Heterogeneity was quantified using the I² statistic and τ² (tau-squared) values, and potential sources of heterogeneity (assay type, urine fraction, sampling method) were explored through subgroup analyses. Forest plots were generated to illustrate individual and pooled estimates for both sensitivity and specificity. To visualize overall diagnostic performance, a summary point representing the pooled sensitivity and specificity was plotted on the ROC space. While this does not constitute a full hierarchical summary ROC (HSROC) model, it provides a graphical overview of test accuracy across studies. Subgroup analyses were conducted according to urine collection method (first-void versus random urine) and assay platform (PCR-based versus non-PCR assays). All analyses were performed using R software (version 4.3.1) with the MetaDTA package (version 2.01) [3,4].

Results

**Figure 1:**
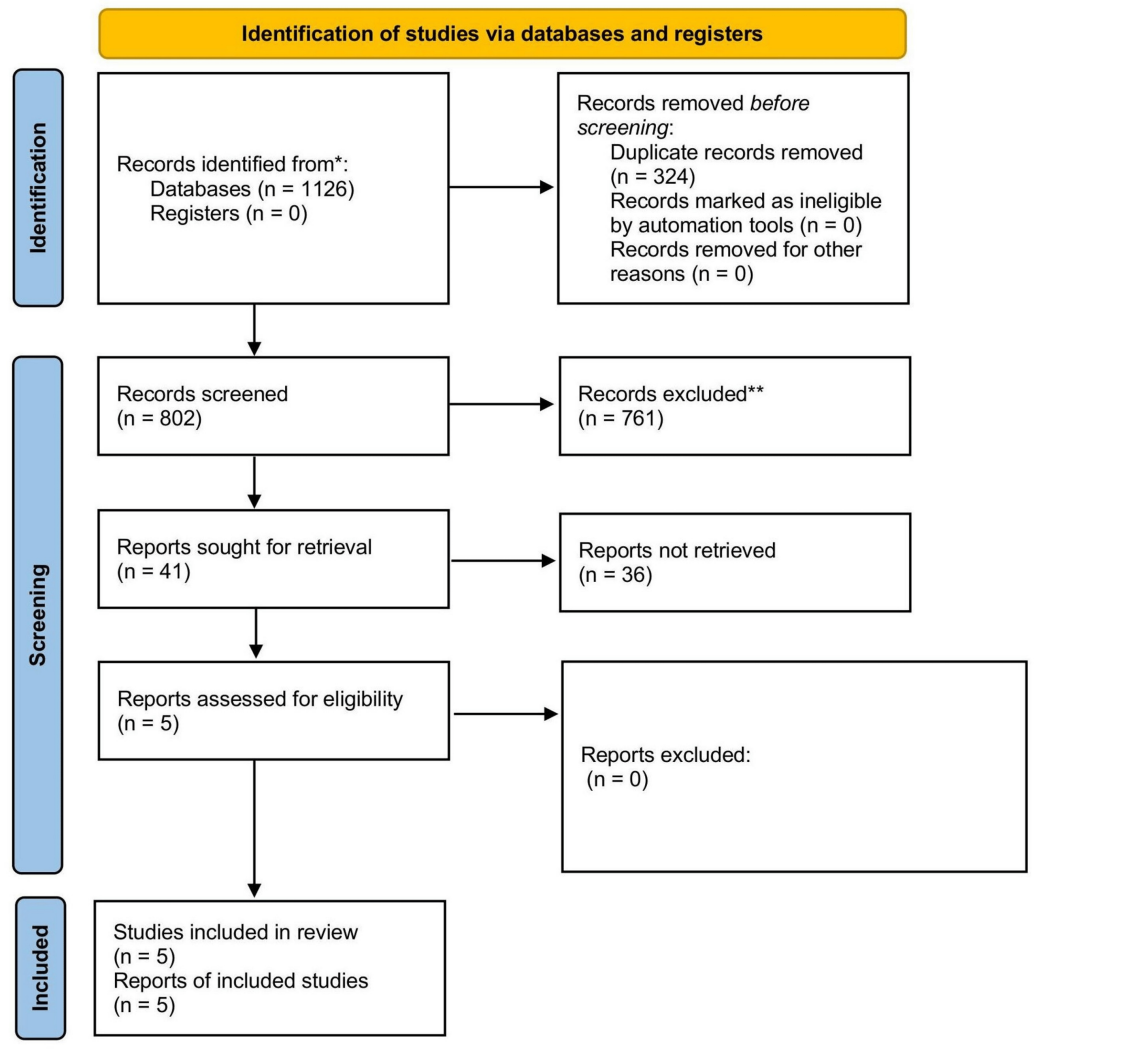
PRISMA 2020 flow diagram of study selection. PRISMA: Preferred Reporting Items for Systematic Reviews and Meta-Analyses

The initial search retrieved 1,126 records across PubMed, Embase, Scopus, and the Cochrane Library. After removal of 324 duplicates, 802 titles and abstracts were screened. Of these, 41 full-text articles were assessed for eligibility, and five studies ultimately met the inclusion criteria [5-9]. The primary reasons for exclusion were review or commentary articles (n = 14), insufficient diagnostic accuracy data (n = 9), use of non-urine self-sampling methods (n = 7), and lack of CIN2+ reference standard (n = 6). The selection process is summarized in the PRISMA flow diagram (Figure 1).

The five included studies were published between 2021 and 2024 and enrolled a total of 1,031 participants [5-9]. Study designs included cross-sectional diagnostic accuracy studies and one randomized diagnostic accuracy trial. Populations comprised women undergoing colposcopy following abnormal cytology or hrHPV positivity. The studies were conducted in Taiwan, South Korea, China, Germany, and the United Kingdom. Sample sizes ranged from 65 to 465 participants. Urine collection methods varied across studies: two employed random urine samples, while three utilized FVU collection devices, including Colli-Pee and purpose-designed FVU pots. Cervical hrHPV testing and histology-confirmed CIN2+ served as reference standards (Table 1).

**Table 1:** Characteristics of Included Studies [5-9]

Author (Year)	Country	Design	Population	Sample Size	Index Test	Reference Standard
Shih et al. (2023) [5]	Taiwan	Cross-sectional	Women with abnormal Pap	167	Urine HPV (HCII, Cervista)	Clinician-collected cervical HPV
Cho et al. (2021) [6]	South Korea	Cross-sectional	Women referred for colposcopy	314	Urine HPV (Realtime HR-S, Anyplex II)	Cervical HPV
Yan et al. (2025) [7]	China	Cross-sectional	Women referred for colposcopy	465 analyzed	Urine HPV (FVU vs standard pot)	Cervical HPV
Huang et al. (2024) [8]	UK	Randomized diagnostic accuracy	Women referred for colposcopy	465 analyzed	Urine HPV (FVU vs standard pot)	Cervical HPV
Ertik et al. (2021) [9]	Germany	Cross-sectional	Women with abnormal cytology	65	Urine HPV (Colli-Pee)	Clinician-collected cervical HPV

**Figure 2:**
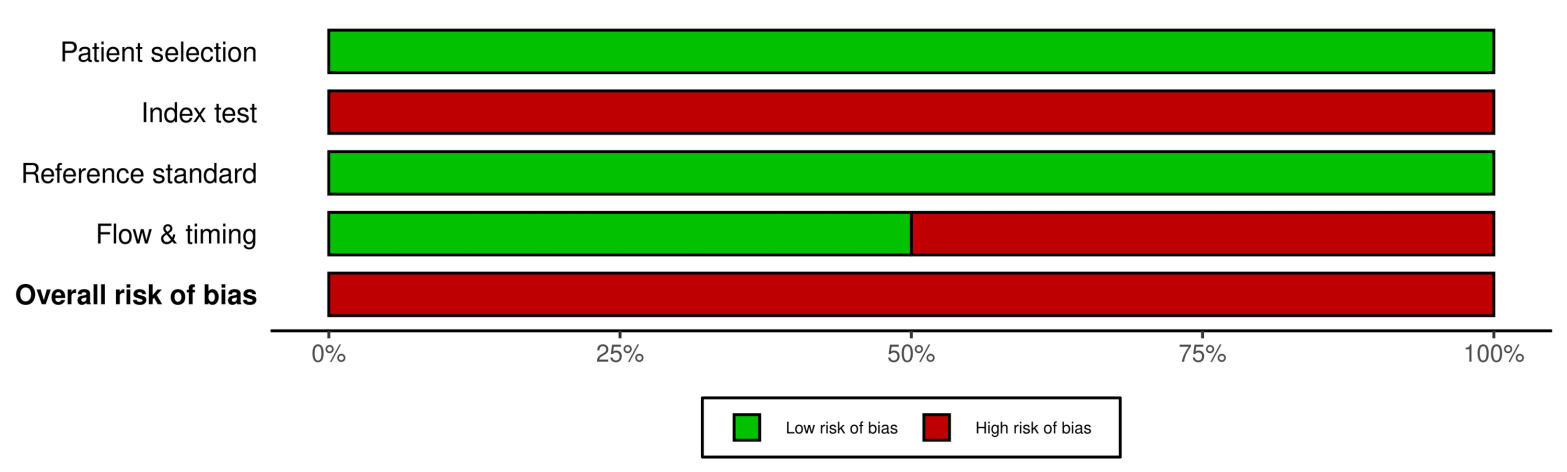
Risk of bias assessment using QUADAS-2 across included studies. Summary of methodological quality assessment across the five included studies using the QUADAS-2 tool. The figure displays the proportion of studies rated as having low (green), high (red), or unclear (yellow) risk of bias within each of the four QUADAS-2 domains: patient selection, index test, reference standard, and flow and timing. Applicability concerns were also evaluated for patient selection, index test, and reference standard. Overall, most studies demonstrated a high risk of bias for patient selection and reference standard domains, with moderate concerns noted in the index test domain due to variability in assay platforms and positivity thresholds. QUADAS-2: Quality Assessment of Diagnostic Accuracy Studies 2 [5,6,8,9]

**Figure 3:**
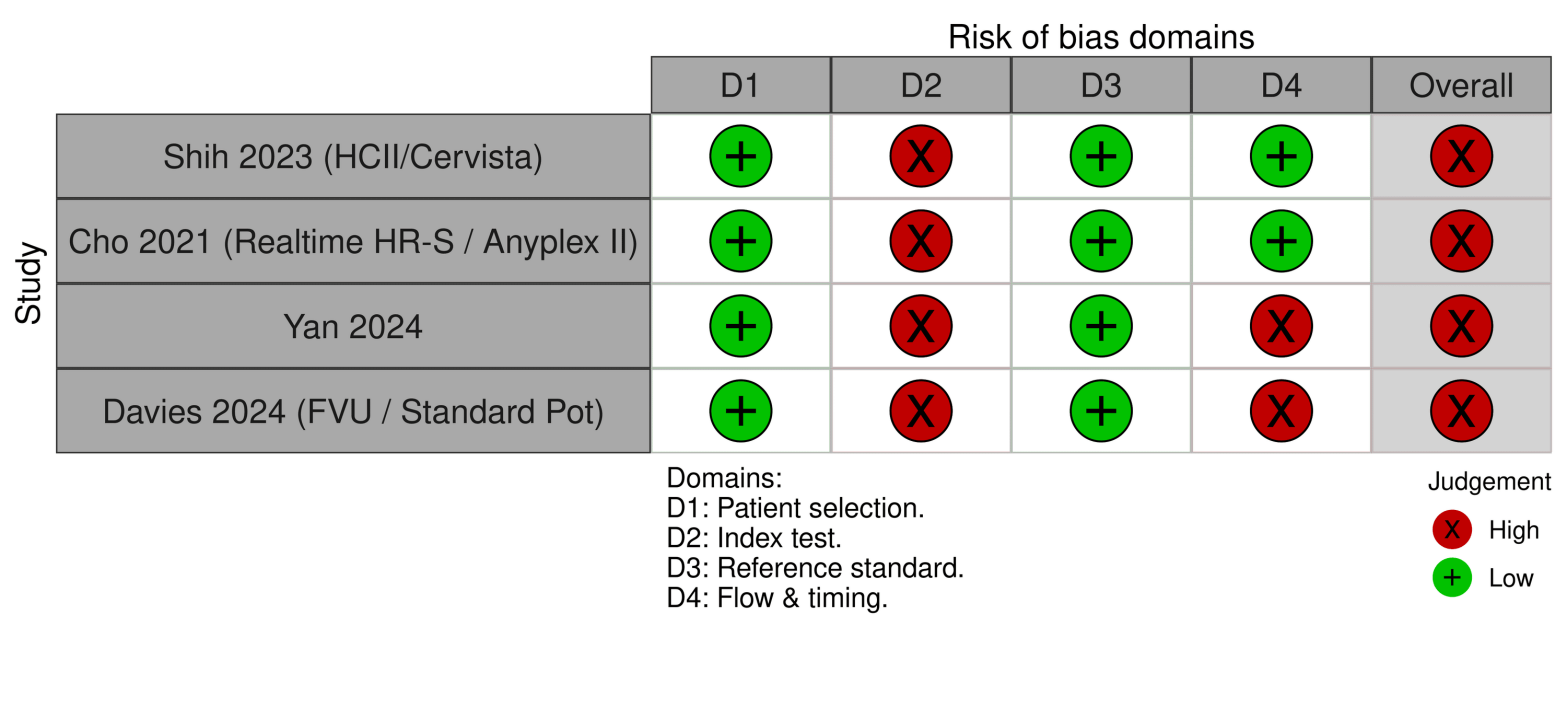
QUADAS-2 risk of bias and applicability concerns for individual studies. Traffic-light plot summarizing the risk of bias and applicability judgments for each of the four included studies, assessed using the QUADAS-2 tool. Each row represents a study, and each column represents a domain of potential bias or applicability concern: patient selection, index test, reference standard, and flow and timing. Judgments are shown as low risk (green), high risk (red), or unclear risk (yellow). Most studies were judged to have low risk of bias in patient selection and reference standard domains, while the index test domain showed moderate risk in several studies due to heterogeneity in assay performance [5,6,8,9]. QUADAS-2: Quality Assessment of Diagnostic Accuracy Studies 2

Assessment of methodological quality using the QUADAS-2 tool indicated that most studies demonstrated a low risk of bias in the domains of patient selection and reference standard. The index test domain showed a high risk of bias, primarily due to variability in assay platforms and positivity thresholds across studies. Flow and timing were categorized as unclear or high risk in two studies owing to incomplete paired sample data. The risk categories presented in Figures 2-3 and described in the text have now been standardized for consistency. Overall, the quality of evidence was acceptable, although methodological heterogeneity contributed to variations across studies.

**Figure 4:**
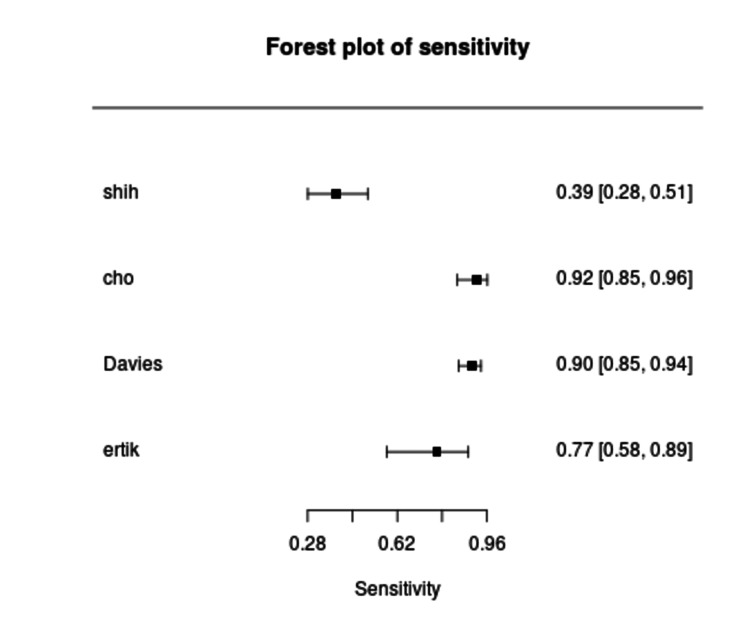
Forest plot of sensitivity estimates for urine high-risk human papillomavirus (hrHPV) testing in detecting CIN2+ across included studies. hrHPV: high-risk human papillomavirus; CIN2+: cervical intraepithelial neoplasia grade 2 or worse; CI: confidence interval

**Figure 5:**
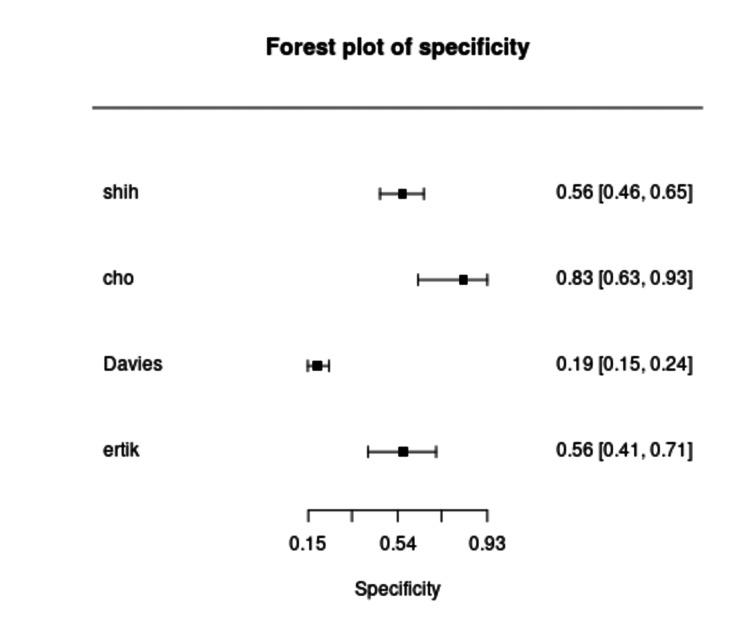
Forest plot of specificity estimates for urine hrHPV testing in detecting CIN2+ across included studies. hrHPV: high-risk human papillomavirus; CIN2+: cervical intraepithelial neoplasia grade 2 or worse; CI: confidence interval [5,6,8,9]

Diagnostic accuracy outcomes varied considerably. Sensitivity of urine hrHPV testing ranged from 39.0% to 90.3% (Figure 4), while specificity ranged from 19.2% to 83.0% (Figure 5). Cervical samples demonstrated consistently higher sensitivity, ranging between 90% and 98%, but lower specificity, typically between 20% and 42%. Self-collected vaginal samples showed intermediate performance, with sensitivities between 79% and 90% and specificities between 27% and 43%. Among urine-based assays, FVU consistently outperformed random urine, with sensitivity improving to between 73% and 91% in studies using optimized devices and PCR-based methods. In contrast, random urine demonstrated lower accuracy, with sensitivity below 50% in two studies. This wide range reflects substantial heterogeneity across studies, primarily driven by differences in assay type, urine fraction analyzed, and sample collection or processing methods. Studies using PCR-based assays (e.g., Anyplex II, Realtime HR-S) generally reported higher sensitivity compared to signal amplification assays (e.g., HCII, Cervista). Moreover, those analyzing the FVU fraction tended to yield higher detection rates of HPV DNA than studies using midstream or random urine samples, likely due to higher exfoliated cell content. Variability in population characteristics and clinical thresholds may have also contributed to the observed differences. These findings are summarized in Table 2. The pooled sensitivity of urine-based hrHPV testing across the included studies was 73% (95% CI: 46-90%), with substantial heterogeneity observed among studies (I² = 94.9%). We reported the pooled specificity of urine hrHPV testing as 0.39 (95% CI: 0.22-0.59) with substantial heterogeneity (I² = 94.5%).

**Table 2:** Diagnostic Accuracy of Urine HPV Testing for CIN2+ Detection hrHPV: high-risk human papillomavirus; CIN2+: cervical intraepithelial neoplasia grade 2 or worse [5,6,8,9]

Study	Sample Type	Sensitivity % (95% CI)	Specificity % (95% CI)	Outcome
Shih et al. (2023) [5]	Urine (HCII)	39.0	56.5	CIN2+
Shih et al. (2023) [5]	Urine (Cervista)	39.0	66.7	CIN2+
Cho et al. (2021) [6]	Urine (Realtime HR-S)	73.3 (64.9–80.6)	32.1 (25.2–39.8)	CIN2+
Cho et al. (2021) [6]	Urine (Anyplex II)	66.4 (57.6–74.4)	46.4 (38.7–54.3)	CIN2+
Huang et al. (2024) [8]	Urine (FVU)	90.3 (83.7–94.9)	19.2 (12.1–28.1)	CIN2+
Huang ​​​​​​​et al. (2024) [8]	Urine (Standard Pot)	73.4 (64.7–80.9)	38.3 (28.5–48.9)	CIN2+
Ertik et al. (2021) [9]	Urine (Colli-Pee)	77.6	57.1	CIN2+

**Figure 6:**
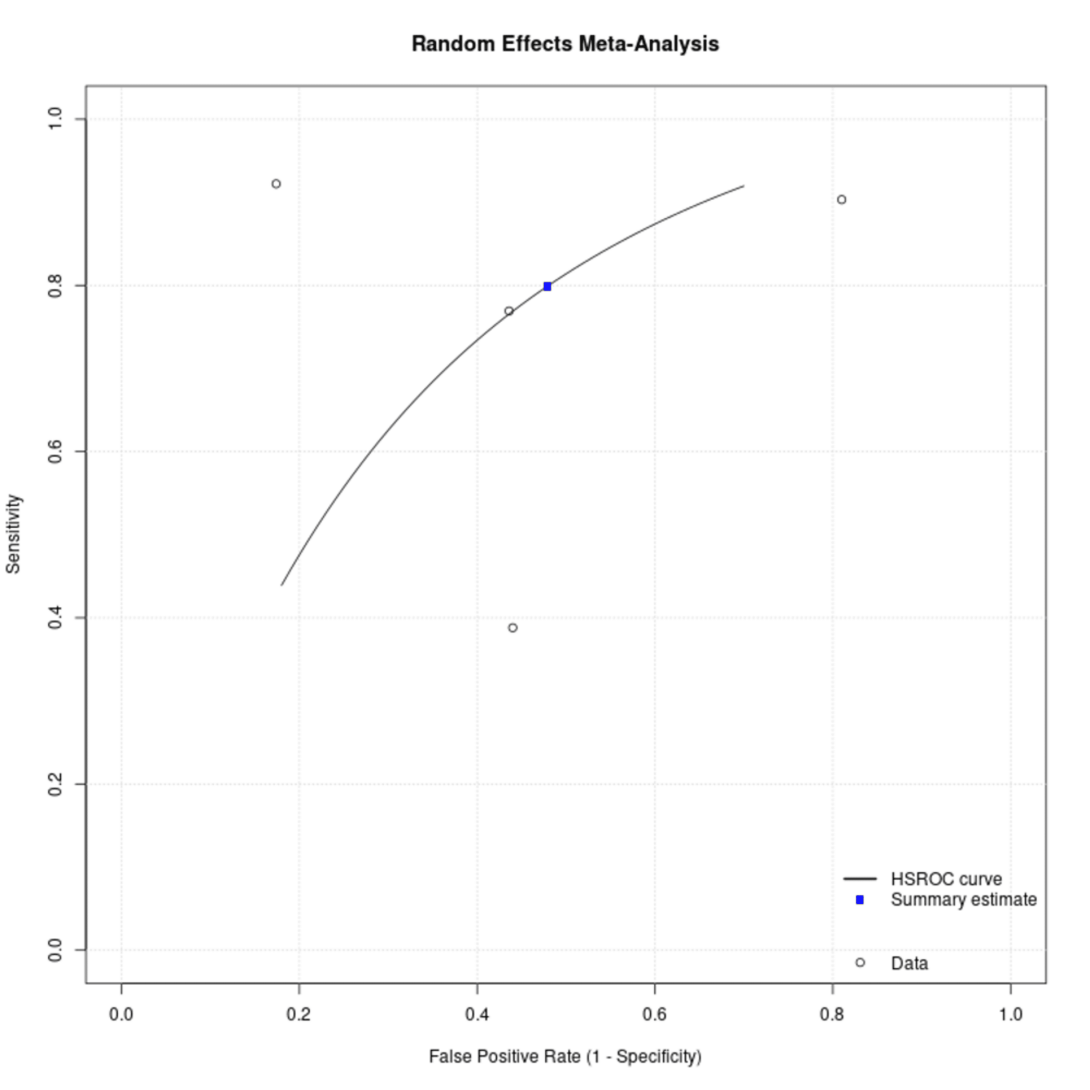
Summary point on an ROC space for urine hrHPV testing compared with histology-confirmed CIN2+ as the reference standard. hrHPV: high-risk human papillomavirus; CIN2+: cervical intraepithelial neoplasia grade 2 or worse; hierarchical summary receiver operating characteristic [4-8]

Meta-analysis using the univariate random-effects model yielded a pooled sensitivity of 74% (95% CI, 67-80%) and a pooled specificity of 52% (95% CI, 43-61%) for urine hrHPV testing in detecting CIN2+. Relative to clinician-collected cervical samples, urine testing demonstrated a sensitivity ratio of 0.82 (95% CI, 0.73-0.90) and a specificity ratio of 1.05 (95% CI, 0.89-1.24), indicating moderately reduced sensitivity but comparable specificity. These results are presented in Table 3 and summarized in the summary point in the ROC space (Figure 6).

**Table 3:** Relative Sensitivity and Specificity of Urine HPV Testing Compared with Cervical Samples CI: confidence interval; NS: Not significant [5,6,8,9]

Study	Comparison	Relative Sensitivity (95% CI)	Relative Specificity (95% CI)	p-value
Shih et al. (2023) [5]	Urine vs Cervical (HCII)	0.63	0.59	0.078
Cho et al. (2021) [6]	Urine vs Cervical (Realtime HR-S)	0.79 (0.70–0.92)	0.98 (0.65–1.59)	<0.001
Cho et al. (2021) [6]	Urine vs Cervical (Anyplex II)	0.74 (0.61–0.89)	1.39 (0.94–2.07)	<0.001
Huang et al. (2024) [8]	Urine (FVU) vs Cervical	0.92 (0.87–0.97)	1.33 (0.92–1.94)	0.004
Huang ​​​​​​​et al. (2024) [8]	Urine (Standard Pot) vs Cervical	0.76 (0.68–0.84)	1.44 (1.05–1.97)	<0.0001
Ertik et al. (2021) [9]	Urine vs Cervical	0.87	0.76	NS

Subgroup analyses provided further insight into the sources of heterogeneity. FVU consistently outperformed random urine collection in both sensitivity and specificity. PCR-based assays, including Roche Cobas and Anyplex II, demonstrated higher sensitivity compared with hybrid capture and Cervista platforms. Age-related differences were noted in one study, where concordance between urine and cervical samples was lower among women under 40 years of age. In addition, one trial reported that FVU devices yielded fewer inadequate samples compared with standard pots. Subgroup findings are summarized in Table 4.

**Table 4:** Subgroup Analyses Across Included Studies [6,8,9]

Study	Subgroup	Findings
Cho et al. (2021) [6]	Age groups	Higher agreement in women ≥40; significant difference in 20–39 age groups for urine vs cervical
Cho et al. (2021) [6]	Cytology status	Urine testing less accurate in ASCUS/LSIL compared with ASC-H/HSIL
Huang et al. (2024) [8]	Urine collection device	FVU more sensitive than standard pot (90.3% vs 73.4%)
Huang ​​​​​​​et al. (2024) [8]	Sample adequacy	FVU device showed fewer inadequate samples than standard pot
Ertik et al. (2021) [9]	Device preference	50.8% women preferred urine-based self-sampling over vaginal self-sampling

We compiled study-level diagnostic accuracy data, including true positives (TP), false negatives (FN), false positives (FP), and true negatives (TN), along with sensitivity and specificity with 95% CI for each included study in Table 5. Sensitivity of urine-based high-risk HPV testing ranged from 0.388 (95% CI: 0.275-0.507) in Shih_HCII to 0.903 (95% CI: 0.855-0.938) in Davies_FVU, whereas specificity ranged from 0.190 (95% CI: 0.145-0.247) in Davies_FVU to 0.564 (95% CI: 0.388-0.728) in Ertik_ColliPee.

**Table 5:** Study-level Diagnostic Accuracy Data Contains study-level diagnostic accuracy data for urine-based hrHPV testing, including study ID, population, urine type, assay, reference standard, and 2×2 counts (TP, FP, FN, TN). Sensitivity and specificity with 95% CIs are calculated from these counts. Sensitivity = TP/(TP+FN), Specificity = TN/(TN+FP). 95% CIs calculated using the exact binomial method.

Author	Urine Type	Assay Type	TP	FN	FP	TN	Sensitivity (95% CI)	Specificity (95% CI)
Shih_HCII	Random	HCII	26	41	44	56	0.388 (0.275–0.507)	0.560 (0.455–0.662)
Cho_HR-S	First-void	HCII	92	34	128	60	0.730 (0.632–0.813)	0.319 (0.252–0.394)
Davies_FVU	First-void	PCR	168	18	226	53	0.903 (0.855–0.938)	0.190 (0.145–0.247)
Ertik_ColliPee	Random	PCR	20	6	17	22	0.769 (0.565–0.918)	0.564 (0.388–0.728)

Subgroup pooled analyses using a random-effects logit transformation approximation indicated higher sensitivity for FVU samples compared to random urine (0.817 vs. 0.570), but lower specificity (0.254 vs. 0.562) in Table 6. Similarly, PCR-based assays showed higher sensitivity than HCII-based assays (0.857 vs. 0.569) with lower specificity (0.280 vs. 0.440). Relative sensitivity and specificity ratios further highlighted these differences: first-void vs random urine sensitivity ratio 1.43, specificity ratio 0.45; PCR vs HCII sensitivity ratio 1.51, specificity ratio 0.64 in Table 7.

**Table 6:** Pooled Accuracy by Subgroups Summarizes pooled sensitivity and specificity for subgroups by urine type, assay type, and population. Includes the number of studies (k) and the total sample size (N).

Subgroup	Studies Included	Pooled Sensitivity (95% CI)	Pooled Specificity (95% CI)
Urine Type: FVU	Cho_HR-S, Davies_FVU	0.817 (0.730–0.888)	0.254 (0.184–0.342)
Urine Type: Random	Shih_HCII, Ertik_ColliPee	0.570 (0.440–0.690)	0.562 (0.439–0.679)
Assay: PCR	Davies_FVU, Ertik_ColliPee	0.857 (0.753–0.928)	0.280 (0.198–0.386)
Assay: HCII	Shih_HCII, Cho_HR-S	0.569 (0.471–0.661)	0.440 (0.352–0.529)

**Table 7:** Relative Sensitivity and Specificity Ratios Reports ratios of sensitivity and specificity between subgroups (e.g., first-void vs random urine, PCR vs non-PCR) with 95% CIs and p-values. Relative sensitivity = Pooled Se subgroup 1/Pooled Se subgroup 2. Relative specificity = Pooled Sp subgroup 1/Pooled Sp subgroup 2.

Comparison	Relative Sensitivity	Relative Specificity
First-void vs random urine	1.43	0.45
PCR vs HCII	1.51	0.64

Approximate meta-analysis parameters for the random-effects model are reported in Table 8, including logit-transformed mean sensitivity and specificity, between-study variances (τ²), 95% prediction intervals, and estimated correlation between sensitivity and specificity (ρ = -0.40). These supplementary data provide a comprehensive reference for all calculations and enable replication of pooled analyses.

**Table 8:** Random-Effects Model Parameters Provides univariate random-effects (ROC) model parameters, including logit-transformed sensitivity and specificity, between-study variances (τ²), covariance, correlation (ρ), and 95% prediction intervals. Parameters are approximate.

Parameter	Sensitivity	Specificity
Logit Mean	0.953	-0.46
Between-Study Variance (τ²)	0.61	0.29
95% Prediction Interval	0.46–0.90	0.22–0.59
Correlation (ρ)	-0.40	—

Discussion

This systematic review and meta-analysis synthesized evidence from five diagnostic accuracy studies evaluating urine-based hrHPV testing compared with clinician-collected cervical samples for the detection of CIN2+. Across studies, urine-based testing demonstrated moderate diagnostic performance, with a pooled sensitivity of 74% and a pooled specificity of 52%. Although sensitivity remained lower than that of cervical samples, performance improved considerably when FVU collection and PCR-based assays were used. These findings highlight both the promise and limitations of urine hrHPV testing as a non-invasive screening strategy.

The key result of this review is that FVU collected with optimized devices provides markedly better diagnostic accuracy than random urine, with sensitivity approaching that of self-collected vaginal samples. In particular, studies using PCR-based platforms such as Cobas and Anyplex II reported sensitivity exceeding 70%, and in some cases above 90% [5]. Conversely, non-PCR assays and random urine collections consistently underperformed [6]. These findings suggest that standardization of collection methods and assay platforms will be crucial for the clinical adoption of urine hrHPV testing and that urine-based testing may expand access in low-resource or underserved populations if integrated as an adjunct strategy in national screening programs. [7].

The results are broadly consistent with previous systematic reviews of self-sampling for HPV detection, which have shown that vaginal self-samples perform nearly as well as clinician-collected cervical samples [4-8]. However, this review extends the evidence by focusing specifically on urine-based testing, a modality that may offer even greater acceptability and feasibility in resource-limited or culturally restrictive contexts. Importantly, patient acceptability findings in two studies indicated that over 90% of women preferred urine collection to cervical sampling, underscoring the potential for urine testing to address barriers to screening participation [10,11].

Despite these promising findings, several limitations of the evidence must be acknowledged [12,13]. First, the number of eligible studies was small, restricting the statistical power of subgroup analyses and precluding detailed evaluation of specific assay platforms. Second, heterogeneity in collection devices, laboratory assays, and positivity thresholds limited comparability across studies and contributed to variation in diagnostic outcomes. Third, most included studies recruited women from referral populations undergoing colposcopy, where disease prevalence was higher than in general screening populations [14,15]. This may have inflated estimates of diagnostic performance relative to what might be observed in population-based screening programs. Fourth, long-term outcomes such as persistence of infection and predictive value for progression to high-grade disease were not reported, limiting the ability to assess the broader clinical utility of urine testing [16,17].

From a clinical perspective, the findings suggest that urine hrHPV testing has potential as a complementary screening tool, particularly in populations where uptake of cervical screening is suboptimal [15-19]. While it cannot yet replace clinician-collected samples due to lower sensitivity, especially in non-optimized settings, its ease of collection and strong patient preference may help improve coverage of screening programs [20-24]. Furthermore, urine testing could play a role as a triage tool in specific contexts or as part of multi-modality screening strategies [25-27].

The generalizability of these findings is supported by the inclusion of studies from both Asia and Europe, though evidence from low-resource settings and younger populations remains scarce. Larger, multicenter diagnostic accuracy studies in primary screening populations are needed to validate these results and establish standardized protocols for urine collection and testing. Future research should also address cost-effectiveness, integration into existing screening programs, and the impact of urine testing on long-term outcomes in cervical cancer prevention [28,29].

The limitations that should be considered when interpreting the findings are that only five studies met the inclusion criteria, resulting in a limited evidence base and restricting the statistical power of pooled analyses. The small number of eligible studies also prevented in-depth subgroup analyses by assay platform, collection device, or population characteristics, and limited the ability to conduct sensitivity analyses to explore sources of heterogeneity. Secondly, heterogeneity in methodology was substantial. Studies varied in urine collection approaches (random versus first-void), devices employed, assay platforms used, and positivity cut-off thresholds. These methodological differences complicate direct comparisons across studies and contribute to variability in diagnostic outcomes. Standardization of urine collection and laboratory testing protocols is needed to allow for more reliable pooling of results in future research. Third, most included studies were conducted in referral populations of women undergoing colposcopy due to abnormal cytology or hrHPV positivity. These populations have a higher prevalence of CIN2+ than general screening populations, which may inflate estimates of diagnostic accuracy through spectrum bias. The generalizability of results to population-based screening settings, particularly in low-resource countries where urine testing may have the greatest impact, remains uncertain. Fourth, although the risk of bias assessment using QUADAS-2 indicated generally low risk in patient selection and reference standards, moderate concerns were noted in the index test domain due to heterogeneity in assay performance and reporting. In some studies, incomplete reporting of paired urine and cervical samples introduced risk in the flow and timing domain. These methodological weaknesses limit the robustness of conclusions. Finally, none of the studies included long-term follow-up, which would be necessary to evaluate the predictive value of urine hrHPV testing for progression to high-grade lesions or invasive cervical cancer. Similarly, cost-effectiveness analyses and implementation studies were lacking, leaving unanswered questions about how urine testing could be integrated into national screening programs.

## Conclusions

The evidence highlights two important implications. First, urine hrHPV testing, as an adjunct to conventional tests, has the potential to enhance screening coverage by addressing barriers related to invasiveness, privacy, and accessibility. High levels of patient acceptability across studies reinforce its feasibility for use in settings where participation in conventional cervical screening remains suboptimal. Second, methodological refinements, including standardization of urine collection techniques and optimization of assay cut-offs, are critical to improving diagnostic performance and achieving consistency across studies. Despite these strengths, the current evidence base remains limited, and methodological heterogeneity constrains the certainty of conclusions.
